# Development of Clinical-Radiomics Nomogram for Predicting Post-Surgery Functional Improvement in High-Grade Glioma Patients

**DOI:** 10.3390/cancers17050758

**Published:** 2025-02-23

**Authors:** Tamara Ius, Maurizio Polano, Michele Dal Bo, Daniele Bagatto, Valeria Bertani, Davide Gentilini, Giuseppe Lombardi, Serena D’agostini, Miran Skrap, Giuseppe Toffoli

**Affiliations:** 1 Neurosurgery Unit, Head-Neck and Neuroscience Department, University of Udine, 33100 Udine, Italy; 2 Experimental and Clinical Pharmacology Unit, Centro di Riferimento Oncologico di Aviano (CRO) IRCCS, Via Franco Gallini, 2, 33081 Aviano, Italy; 3 Neuroradiology Unit, Department of Diagnostic Imaging, University Hospital of Udine, Piazzale Santa Maria della Misericordia 15, 33100 Udine, Italy; 4 Department of Oncologic Radiation Therapy and Diagnostic Imaging, Centro Di Riferimento Oncologico, Via Franco Gallini, 2, 33081 Aviano, Italy; 5 Department of Brain and Behavioral Sciences, University of Pavia, 27100 Pavia, Italy; 6 Bioinformatics and Statistical Genomics Unit, Istituto Auxologico Italiano IRCCS, Cusano Milanino, University of Milano-Bicocca, 20095 Milan, Italy; 7 Medical Oncology 1, Veneto Institute of Oncology-IRCCS, Via Gattamelata 64, 35128 Padua, Italy

**Keywords:** glioma grade 4 (GG4), machine learning, prognosis prediction, radiomics, precision medicine, web-based prediction tools

## Abstract

**Introduction:** Glioma Grade 4 (GG4) tumors, which include both IDH-mutated and IDH wild-type astrocytomas, are the most prevalent and aggressive form of primary brain tumor. Radiomics is gaining ground in neuro-oncology. The integration of this data into machine learning models has the potential to improve the accuracy of prognostic models for GG4 patients. Karnofsky Performance Status (KPS), an established preoperative prognostic factor for survival, is commonly used in these patients. In this study, we developed a nomogram to identify patients with improved functional performance as indicated by an increase in KPS after surgery by analyzing radiomic features from preoperative 3D MRI scans. **Methods:** Quantitative imaging features were extracted from the -3D T1 GRE sequence of 157 patients from a single center and were used to develop the machine learning (ML) model. To improve applicability and create a nomogram, multivariable logistic regression analysis was performed to build a model incorporating clinical characteristics and radiomics features. **Results:** We labeled 55 cases in which KPS was improved after surgery (35%, KPS-flag = 1). The resulting model was evaluated according to test series results. The best model was obtained by XGBoost using the features extracted by pyradiomics, with a Matthew coefficient score (MCC) of 0.339 (95% CI: 0.330–0.3483) in cross-validation. The out-of-sample evaluation on the test set yielded an MCC of 0.302. A nomogram evaluating the improvement of KPS post-surgery was built based on statistically significant variables from multivariate logistic regression including clinical and radiomics data (c-index = 0.760, test set). **Conclusions:** MRI radiomic analysis represents a powerful tool to predict postoperative functional outcomes, as evaluated by KPS.

## Simple Summary

Karnofsky Performance Status (KPS) is a well established pre-surgical prognostic factor for the survival of patients affected by Glioma Grade 4 (GG4) tumors, including astrocytoma IDH-mutant grade 4 and the astrocytoma IDH wild type. The aim of this study is to develop a nomogram to identify patients with KPS improvement after surgery, starting by using a machine learning approach for the radiomic features extracted from the preoperative -3D T1 GRE sequence of 157 GG4 patients. We labeled 55 cases in which KPS was improved after surgery (35%, KPS-flag = 1). The best model was obtained by XGBoost with a Matthew coefficient score (MCC) of 0.339 (95% CI: 0.330–0.3483) in cross-validation. A nomogram evaluating the improvement of KPS post-surgery was built based on statistically significant variables from multivariate logistic regression including clinical and radiomics data (c-index = 0.760, test set). Thus, MRI radiomic analysis represents a powerful tool to predict postoperative functional outcomes, as evaluated by KPS.

## 1. Introduction

Despite recent advancements in surgery and genetics, Gliomas Grade 4 (GG4), including astrocytoma IDH-mutant grade 4 and the astrocytoma IDH wt, face a grim prognosis, marked by a high likelihood of early tumor recurrence (TR) after postoperative radio-chemotherapy [1]. Glioblastoma (GBM), the most aggressive primary brain tumor among GG4, exhibits a median overall survival (OS) of just 14.6 months [1,2,3,4]. Compelling evidence supports the extent of resection (EOR), derived by objective tumor volume analysis, as a key predictive survival factor, useful in the management of GG4 patients [5,6,7].

Achieving radical surgical treatment is however challenging due to the tumor’s infiltrating nature, multifocal presentation, and poorly defined margins [8].The functional status after resection, quantified by the Karnofsky Performance Status (KPS), represents a crucial metric in evaluating patients’ capacity to endure invasive and intensive therapeutic interventions. KPS plays an important role in understanding the overall patient’s functional capacity, and, as a result, contributing to the postoperative decision-making process [9,10,11,12]. A low value of postoperative KPS can effectively limit these patients’ eligibility for adjuvant postoperative treatments, leading them to extremely rapid disease progression [13]. Overall, postoperative KPS is influenced by surgery and patient age; tumor size and location are the risk factors for increased postoperative complications [11,12,13,14]. 

Moreover, the increase in time to surgery (TTS) for patients with imaging concerning GBM did not impact clinical outcomes and suggests that there was no effect on specific growth rate, KPS, postoperative deficits, survival, discharge location, or hospital length of stay [15,16]. Modern glioma treatment relies heavily on clinical imaging and precise segmentation and plays a crucial role in the development of personalized medicine. However, the challenge lies in intratumoral heterogeneity and limited access to brain tumor tissue.

Magnetic resonance imaging (MRI) is the gold standard for the diagnosis and monitoring of glioma [17,18,19,20,21]. In this context, MRI is considered the most promising candidate to support decision-making in clinical practice, as it is non-invasive and can characterize the heterogeneity of GG4 and differentiate GG4 from other entities through the use of radiomics [20,21,22].

Radiomics is a process that extracts and analyses a lot of quantitative data from medical images that cannot be seen and evaluated with human vision alone [23,24,25].

Using selected radiomic features, radiomics encompasses a range of computational approaches, including machine learning (ML) algorithms, to analyze data to improve diagnostic, cognitive, and predictive accuracy [26], which have shown remarkable performance in predicting outcomes for a variety of diseases in this field. 

In the present study, with the aim to support clinicians in their decision-making process, we started using an ML model to define a way to stratify patients and categorize the risk of postoperative functional outcomes, as defined by KPS, in newly diagnosed GG4. Then, by using a multivariate logistic regression analysis, a clinical-radiomics model was built by incorporating clinical characteristics and radiomics features.

## 2. Materials and Methods

### 2.1. Study Population

The study included 157 patients who underwent surgical resection of a newly diagnosed GG4 (according to the WHO 2016 classification of brain tumors) at the Neurosurgery Department of Udine Hospital between 2014 and 2019. Patients were enrolled in the case–cohort according to the following inclusion criteria: age ≥ 18 years; no previous surgery; no preoperative chemo- or radiotherapy; objective evaluation of preoperative tumor volume on MRI images (3D T1 gradient-Echo imaging ((3D T1 GRE))); and MGMT promoter methylation and IDH1/IDH2 mutation status assessment.

Several key variables were integrated into the clinical, imaging, and histopathological data, including age, sex, pre- and post-surgery Karnofsky Performance Status (KPS), tumor location, tumor laterality, and therapy treatment. Cases were excluded from the case–cohort if one or more of the following criteria were present: incomplete imaging data, follow-up interruption, needle biopsy, previous treatments, and multicentric tumors. Written informed consent was obtained for surgery. Patients provided informed consent in accordance with the local institutional review board requirements and the Declaration of Helsinki. After surgery, all patients underwent Stupp protocol [2], and were included in a study approved by the local Ethics Committee (protocol N. 0036566 /P/ GEN/EGAS, ID study 2538,approved on the 13 November 2018). We adhered to CheckList for EvaluAtion of Radiomics research (CLEAR) [27].

### 2.2. Defining the Kps-Flag 

To identify patients who demonstrated an improvement in performance status following surgery, we developed a decision tool incorporating the KPS-flag. For each case, we calculated the difference between the Karnofsky Performance Status (KPS) after surgery (KPS-POST) and before surgery (KPS-PRE). Patients were classified as having an improved performance status if they met two criteria: (1) an increase in the KPS difference (KPS-POST—KPS-PRE) of at least 10%, and (2) a KPS-POST greater than 80%. These patients were assigned a KPS-flag = 1.

### 2.3. MRI and Preprocessing

All patients underwent MRI examinations prior to surgery. Three-dimensional T1-weighted MRI sequences obtained post-Gadolinium administration (3D T1 GRE) were 3D T1 GRE acquired in the routine clinical workup alternately using two different 1.5 T or a 3.0 T MR Scanners (1.5 Tesla Magnet, Avanto, Siemens, Erlangen, Germany; 1.5 Tesla Magnet, Aera, Siemens, Erlangen, Germany; 3.0 Tesla Magnet, Achieva, Philips Medical System, Best, The Netherlands). The three-dimensional segmentation was conducted by one neuroradiologist (D.B.) with 10 years of experience who manually picked areas of interest (ROI) around the tumor’s margin to capture the complete tumor volume in each slice on 3D T1 GRE using the open-source software ITK-SNAP (www.itk-snap.org, accessed on 15 April 2022) and then was confirmed by an experienced neuroradiologist (S.D.). The flowchart and the workflow of the research are reported in Figure S1A–C.

All MRI data underwent image resampling to isotropic voxels (1 × 1 × 1 mm) with linear interpolation, and intensity normalization (Z-score) before feature extraction. Then, the Pyradiomics package [28] was used to extract radiomics features from each ROI based on its three-dimensional region of interest (3D ROI) [29,30]. 

EOR, MRI index, and volumetric analysis were performed as previously described [29,31]. All procedures followed the recommendations of the Image Biomarker Standardization Initiative (IBSI), which provides recommendations and definitions for the standardization of image biomarker extraction, imaging protocols, and reporting the results [32].

### 2.4. Feature Selection Rad-Model

The feature selection of characteristics was applied due to the high number of variables in the data sets used in comparison to the number of cases [25,33]. Specifically, to select reliable prognostic variables, correlated characteristics were first removed by calculating the Pearson correlation matrix, whereby highly correlated characteristics (ρ > 0.95) were removed. Further information is reported in the Supplemental Material and Methods [34,35].

### 2.5. Machine Learning Approach for Rad-Model Training and Testing

ML models were developed using a three-way holdout method, with stratification based on the KPS-flag, according to previous studies [36,37,38].

For the selection of an initial classification model, we evaluated the performance of an Xtreme Gradient Boosting (XGBoost) and Random Forest (RF) in python 3.10 using xgboost [34] and sklearn library [39]. To interpret the model’s SHAP (Shapley Additive Prediction), the values were computed using TreeExplainer from the SHAP library. Feature importance was ranked based on mean absolute SHAP value across the data set [40]. Further information is reported in the Supplemental Material and Methods. 

### 2.6. Developing Clinical-Radiomic-Model

Clinical characteristics such as sex, age, and basal hypertension were studied. Two authors (D.B. and T.I.) evaluated the semantic radiological characteristics, assessing the following tumor features: Necrotic/Cystic component (yes or no), necrosis (yes or no), Midline Shift (yes or no), ependyma involvement (yes and no), tumor location, and pre- and postoperative tumor volume. Only cases with complete data matrix values were employed.

All pre- and postoperative tumor segmentations were performed manually across all 3D T1 GRE MRI slices on whole tumors, as previously described [29].

Logistic regression was used to develop a clinical–radiological model by integrating the clinical and radiological variables selected by implementing the information detected by the ML approach. Univariate and multivariate analyses were performed to determine the independent predictors by selecting variables (*p* < 0.05). 

A nomogram was constructed using the combined clinical and radiomics model by multivariate logistic regression, as previously described [41]. The C-index and AUC-ROC were calculated to assess the discrimination performance of the radiomics nomogram. In evaluating the calibration of the nomogram, we created a calibration curve that serves as a scatter plot and represents the probability of actual occurrence versus predictions. In addition, the results of the Hosmer–Lemeshow goodness-of-fit test were considered in the calibration assessment to provide a comprehensive assessment of the model’s performance and reliability. The Hosmer–Lemeshow test was used to assess the fitness of the nomogram (*p* > 0.05 indicating good fit). The training and validation tests were applied for all the models. Decision curve analysis (DCA) was used to investigate the clinical utility of the nomogram in both the training and validation sets by using the rmda R package [42,43]. An interactive web-based dynamic nomogram application was constructed with the “DynNom” R package (version 5.0.1) and Shiny website (www.shinyapps.io).

## 3. Results

### 3.1. Study Population

The most important clinical and radiological features of the cases included are listed in Table S1. According to their clinical preoperative diagnosis, the included study cohort comprised 157 GG4 patients for whom complete preoperative MRI data (3D T1 GRE) were available. All patients underwent neurosurgical treatment as previously described [29]. The significant associations among the prognostic parameters are reported in Table S2.

MGMT methylation status was associated with EOR (*p* = 0.042). The localization was associated with ependyma involvement (*p* < 0.001), the shift (*p* = 0.004), and the preoperative volume (Volume MRI-T2, *p* = 0.011). Furthermore, cases that after surgery had a KPS greater than 90 showed an association with the involvement of the ependyma (*p* = 0.003) and the average hospital length of stay (*p* < 0.001) as expected (Table S2).

### 3.2. Constructing a Machine Learning Model of the Radiomics Signature 

**Figure 1:**
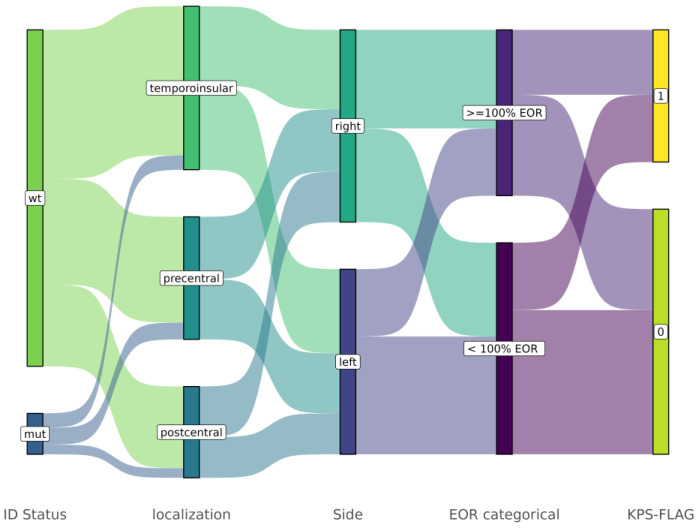
A Sankey diagram to visualize the flow and relationships between categorical variables created based on the relationship among localization, EOR, and flag to predict KPS improvement. The width of the flow represents the proportion of patients moving from one category to another. Of note, GG4 cases labeled with a KPS-flag = 1 showed an improvement of performance status by a heterogeneity of localization, side, and different rates of EOR. For these reasons, we evaluate the informative effect of radiomics data to classify that condition (Figure 1).

To develop the prognostic model, we calculated the difference in KPS before and after surgery. We found 55 cases (35%) with a KPS-flag = 1 (details in Material and Methods) (Table S1). In particular, the association among the molecular information (IDH mutation status) and the tumor localization, side, and extent of resection is shown by the Sankey diagram of Figure 1.

A total of 1132 features were extracted from 3D T1 GRE images using Pyradiomics, including shape (14 features), first-order intensity statistics (18 features), Gray Level Co-occurrence Matrix (22 features), Gray Level Size Zone Matrix (16 features), Gray Level Run Length Matrix (16 features), Gray Level Dependence Matrix (14 features), logarithm (344 features), and wavelet (688 features).

**Figure 2:**
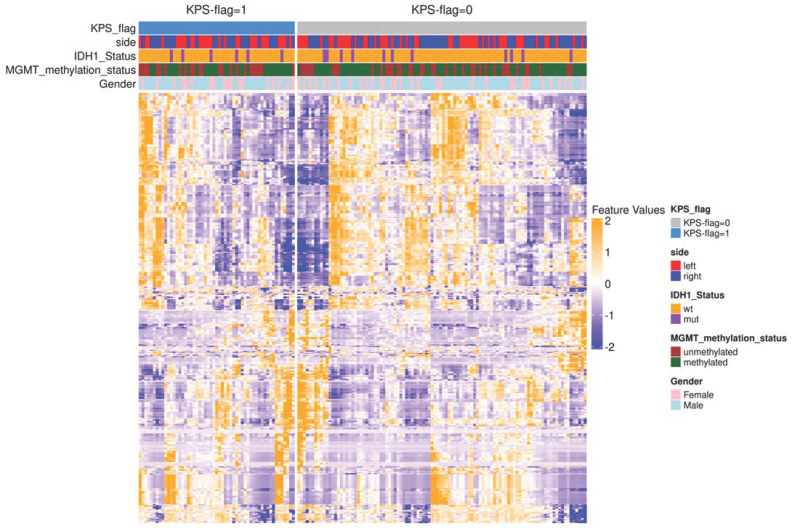
A unsupervised clustering heatmap of the radiomic features extracted from 157 GG4 cases using PyRadiomics. Each row represents a radiomic feature, while each column corresponds to a patient case. The features are standardized using z-score normalization, and hierarchical clustering was performed using Euclidean distance and Ward’s linkage method. The top annotation bar includes key clinical and molecular features such as FLAG status, IDH mutation status, MGMT methylation status, tumor laterality, location, and additional clinical data. This visualization highlights potential patterns and subgroup structures within the radiological landscape of the GG4 cases.

Figure 2 shows the landscape of radiomic features extracted from 3D T1 GRE images. In the rows, the cases are shown and, in the columns, the extracted quantitative features by Pyradiomics are shown. It is noteworthy that the unsupervised clustering did not reveal a precise pattern associated with the KPS-flag present in the data.

Given the large number of features extracted from MRI segmentation, several feature selection procedures were applied to determine the optimal set of features as described in the Supplemental Material and Methods. No significant differences were found between the clinical variables in the training and test group (Table S3).

To test the association between a KPS-flag = 1 and the preoperative radiomics (3D T1 GRE), two ML algorithms were used: Random Forest (RF) and Extreme Gradient Boosting (XGBoost). For both approaches, we used Bayesian optimization to adjust hyper-parameters to obtain the best area under the Receiver Operating Characteristic Curve (ROC-AUC) and Matthew’s correlation coefficient (MCC).

The XGBoost achieved a mean cross-validation MCC of 0.339 (95% bootstrapped confidence interval: 0.330–0.3483) using 174 features, which is significantly higher than the RF model with a mean cross-validation MCC of 0.104 (−0.07–0.26) (Kruskal–Wallis *p* = 0.034). On the test set, an out-of-sample MCC of 0.302 was obtained (Table 1).

**Figure 3:**
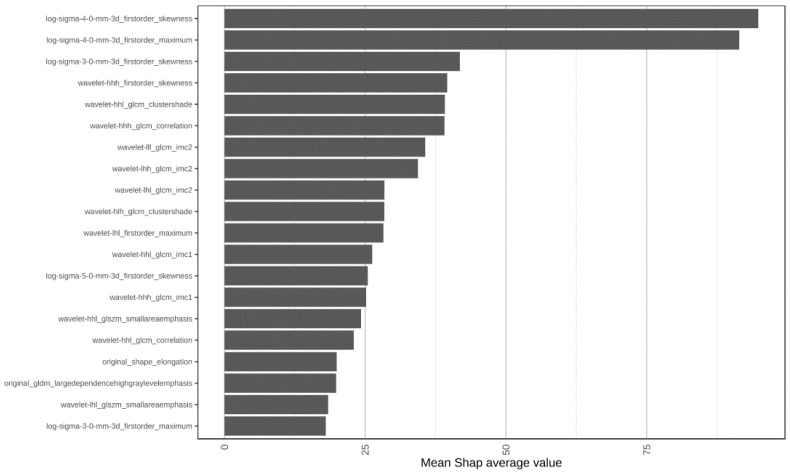
The top 20 feature variables and their importance in the FLAG-KPS model. The figure illustrates the top 20 most influential features identified by the FLAG-KPS model, ranked by their importance scores. The importance was determined using the average SHAP (Shapley Additive Explanations) values over 1000 bootstrap model iterations. Higher SHAP values indicate greater influence on model predictions and provide insight into the key factors driving the model’s decision-making process.

Furthermore, we evaluated the model with a random labels experiment on the same data and found a deterioration in the metrics compared to our best performing model, demonstrating the validity and effectiveness of our model preparation. The radiomic features ranked by SHAP analysis are shown in Table S4. The first 20 most important radiomic features by using 3D T1 GRE for predicting the patients with improved performance status labeled by KPS-flag = 1 are displayed in Figure 3. In detail, the included features are derived from first-order statistics, Gray Level Co-occurrence Matrix (GLCM), and Wavelet Transform features. The top four features extracted are features such as skewness at different spatial scales and maximum intensity values (log-sigma-4-0-mm-3d_firstorder_skewness, log-sigma-3-0-mm-3d_firstorder_skewness), which offer valuable insights into tissue characteristics, pathological conditions, and regions of interest, aiding in diagnostic interpretation and treatment planning (Figure 3). Of note, the skewness reflects deviations from a normal distribution that may indicate changes in tissue composition such as necrosis, edema, or hemorrhage. Higher skewness values may indicate the presence of necrotic or cystic regions that have an uneven intensity distribution due to fluid accumulation or degraded tissue. In this context, the log-sigma-3-0-mm-3D_firstorder_skewness metric shows a significant difference between the cohort of Necrotic/Cystic patients with improved KPS (*p*-value < 0.05) (Figure S2).

**Figure 4:**
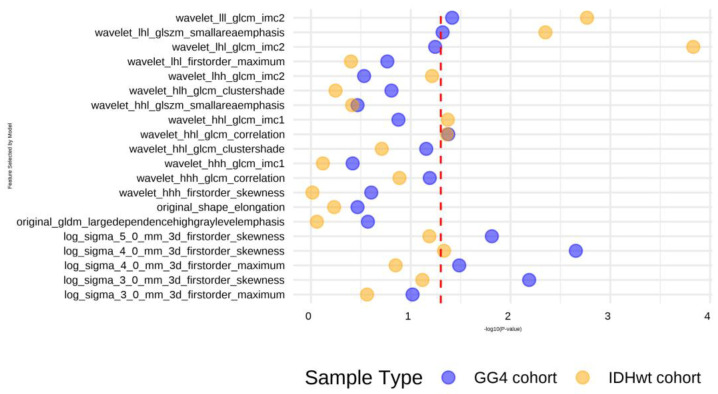
A comparison of the top 20 radiomic features selected by the XGBoost model with the Wilcoxon signed-rank test between the GG4 patient cohort and the IDH wild-type (IDHwt) patient subgroup classified according to the 2021 WHO guidelines. The Wilcoxon signed-rank test is used to detect statistically significant differences in the distribution of radiomic features between these groups identified by the xgboost model. The results are presented in a log10-transformed *p*-value plot highlighting the most important features based on their statistical significance. This analysis sheds light on the radiomic features that differentiate the GG4 cases from the IDHwt subgroup and can thus contribute to classification and prognostic assessment.

In an attempt to address the potential limitations posed by the use of the GG4 cohort [5], we extended our evaluation to include IDH-wild type (IDHwt) samples during model development. Notably, in the context of the IDHwt subgroup, only six patients demonstrated an improvement, as defined by our criteria. To enhance the model’s performance, we applied the Synthetic Minority Over-sampling Technique (SMOTE) algorithm to create a balanced subgroup. However, the results from this approach revealed significant overfitting, as evidenced by high metrics (MCC = 0.90; AUC = 0.91). Further investigation was conducted to assess the selected features using the Wilcoxon rank sum test in the context of the IDHwt cohort. The analysis suggested that certain features exhibit distinct distributions within the IDHwt cohort, implying potential variability in their relevance or predictive power for this subgroup (Figure 4). 

### 3.3. Performance of Clinical–Radiological Model and Nomogram

**Figure 5:**
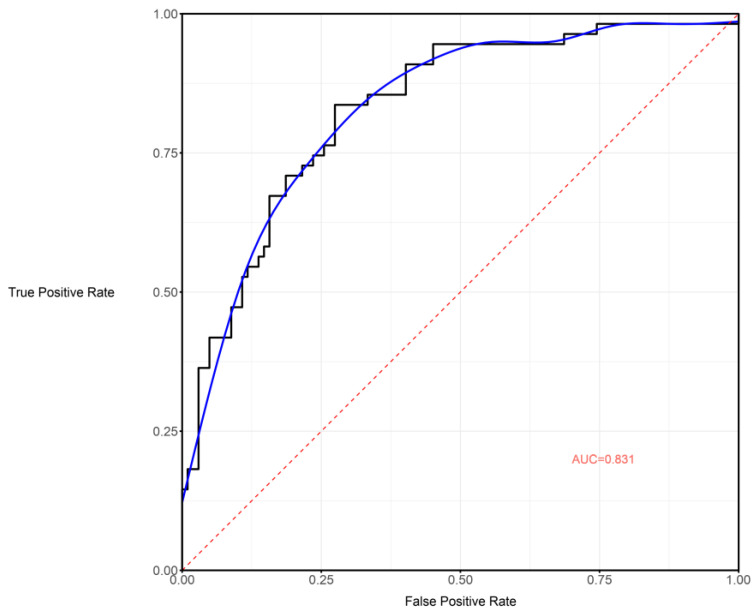
Receiver Operating Characteristic (ROC) curve of the clinical-radiomics nomogram model. The ROC curve illustrates the model’s ability to distinguish between outcome classes by plotting the true positive rate (sensitivity) against the false positive rate (1—specificity) across different probability thresholds. The area under the curve (AUC) was 0.823 (95% CI: 66.1–96.41%), computed using 100 stratified bootstrap iterations. A higher AUC indicates stronger discriminative performance, highlighting the model’s effectiveness in clinical decision-making and predictive accuracy.

**Figure 6:**
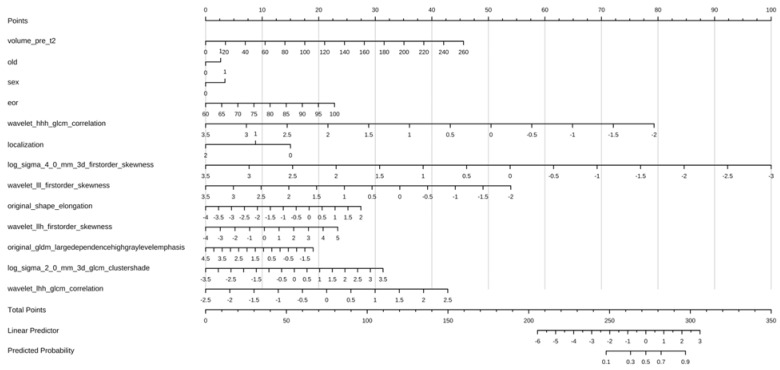
A clinical-radiomics nomogram. The clinical-radiomics nomogram developed for the GG4 cohort by integrating a multivariate logistic regression model constructed using the rms package. This nomogram combines both clinical and radiomic features to provide a quantitative tool for individual risk assessment and outcome prediction. By assigning a weighted contribution to each predictor, the model facilitates the intuitive interpretation of complex relationships among the variables. The use of the rms package ensures robust model calibration, validation, and visualization and increases the reliability and clinical applicability of the nomogram.

A nomogram was obtained by collecting the main radiomic features from the ML model (Table S4) and combining them with the clinical variables. Then, the variables that were determined to be significant by univariate logistic regression were included in the multivariate analysis and, after stepwise selection, the final model was categorically adjusted for age, gender status, and extent of resection (Hosmer and Lemeshow goodness of fit *p* = 0.852, AUC 0.823, 95% CI: 66.1%–96.41%, 100 stratified bootstrap replicates Figure 5, Figure S3). Complete logistic regression is presented in Table 2. The clinical-radiomics nomogram yielded a C-index of 0.825 and of 0.760 in the train and validation cohorts, respectively. The nomogram showed the same diagnostic efficacy as the radiomics model (Figure 6, Figure S2). The online version of the nomogram established in this study is available on an open-access website (URL: https://radiomlcro.shinyapps.io/KPSGLIO/, accessed on 15 April 2022).

The calibration curves of the nomogram (Figure S4) showed good agreement between the predicted probability and the observed outcomes of the improved performance status in both the training and validation sets, indicating good generalizability (mean absolute error (MSE_train_) *p* = 0.047 for train and (MSE_test_) *p* = 0.00248 for the test set). The analysis of the decision showed that the net benefit of the combined model was higher for almost the entire range of *p*-values (Figure S5).

## 4. Discussion

Recognizing patients at risk of a decline in KPS six months after surgery is crucial for tailoring their comprehensive care plan. This identification holds substantial implications for customizing overall management, aiming to strike a balance between preserving Quality of Life (QoL)—which may be compromised by frequent clinical complications—and implementing a more assertive treatment strategy. KPS stands out as the primary factor in determining the eligibility of patients for postoperative radio-chemotherapy, thus inevitably impacting the overall prognosis [9,44,45]. Here, an ML approach specifically tailored to the assessment of postoperative KPS in patients with GG4 was thus developed. 

The primary benefit of using the ML model over conventional statistics lies in its capacity to grasp the nonlinear impact of predictors. This capability enables the detection of factors that may seem not significant when examined individually but become significant when considered in association with others within the complex and heterogenous clinical setting of GG4 [35,46]. 

Our research addresses this critical issue by utilizing the power of ML to develop a robust and accurate prediction model for 3D T1 GRE. The best result was achieved by the XGBoost model with an out-of-sample MCC of 0.23. These findings support the notion that radiomics features extracted from 3D T1 GRE lesion could contribute to developing a decision model that allows for the identification of patients with improved performance status [19,40,41]. It is noteworthy that recent studies have illustrated the potential of radiomic analysis using 3D T1 GRE in predicting the occurrence of pseudo-progression in high-grade gliomas, suggesting its applicability for other purposes as well [47]. 

Regarding the practical implications of this approach, our model demonstrates the feasibility of a straightforward approach to stratifying patients based on 3D T1 GRE features and clinical data. If validated, this approach could potentially assist in decision-making regarding neurosurgical intervention or serve as a starting point for considering the next phase of the patient’s treatment journey. By providing early stratification, our model could offer clinicians more time to evaluate and decide on the most appropriate therapeutic approach. Importantly, unlike other approaches [48,49], our model relies solely on a limited set of features extracted from 3D T1 GRE imaging, which may offer advantages in terms of interpretability and practical application.

On the other hand, the present study has some limitations, mainly due to the retrospective nature of this investigation and the simple size, that could lead to some bias. Moreover, the analysis of a mono-institutional cohort could challenge the generalizability of results when an external patient population is considered

The model we developed aims to evaluate whether radiomic features, combined with preoperative clinical variables, can predict which patients are likely to experience an improvement in their Karnofsky Performance Status (KPS) following surgery. This approach could potentially guide future patient management strategies, including eligibility for innovative therapeutic clinical trials. However, the small sample size poses a significant limitation. Specifically, only six IDH WT patients showed an improvement in KPS, which precludes generalizable analysis even with the application of synthetic data approaches. Additionally, we were unable to incorporate variables such as molecular characterization, the experience of the neurosurgical team, type of anesthesia, or surgical timing due to a lack of comprehensive data. These factors could provide a more complete understanding of the observed outcomes. Nevertheless, we believe that our exploratory study provides a foundation for estimating the necessary sample size for a future multicenter, prospective observational study. Such a study could more precisely address the research question by incorporating additional sources of information and refining the predictive model. 

Although some previous studies already address the prediction of post-surgical functional status, they differ from the present study in several key aspects. First, these studies focus on predicting baseline or postoperative KPS as a static outcome, rather than identifying patients who are likely to experience a significant improvement in KPS following surgery. Our study, in contrast, specifically targets the prediction of KPS improvement, which represents a distinct and clinically relevant endpoint. Second, the methodologies employed in these studies differ substantially from ours. While they rely on either deep imaging features or resting-state functional MRI (fMRI) combined with machine learning, our approach integrates radiomic features derived from conventional imaging modalities with preoperative clinical variables. This multimodal strategy allowed us to capture a broader spectrum of information that may influence post-surgical outcomes. Additionally, the aforementioned studies are limited to specific imaging modalities—such as T1-weighted imaging or fMRI—whereas here we explored the potential of combining radiomic features from multiple imaging sequences. This provides a more comprehensive assessment of the tumor’s characteristics and their relationship to functional outcomes. Finally, while these studies propose more complex models, their sample sizes are comparable to ours, highlighting the challenges of generalizability in this field [48,49]. Our exploratory study, despite its limitations, lays the groundwork for future research by identifying the need for larger, multicenter studies that incorporate additional variables, such as molecular profiling and surgical factors, to refine predictive models for post-surgical KPS improvement.

The features used in the most powerful model to predict KPS were mainly from three categories: first-order statistics, Gray Level Co-occurrence Matrix (GLCM) features, and wavelet features. These categories included a wide range of descriptors that represent different aspects of image characteristics and improve the predictive ability of the model in different contexts [50,51]. 

Nomograms serve as graphical tools to estimate the probability of a particular outcome and rely on several predictor variables, using geometric diagrams to visually represent the results of the predictive model [46,52,53]. Many authors created radiomics models for classification purposes of glioblastoma [23,38,41], in particular, for differentiating glioblastoma from brain metastasis [13,22,44] or for predicting the molecular status of IDH1 [54,55,56]. To obtain a nomogram, we decided to integrate the information from the ML model together with selected clinical data in a logistic regression to identify the cases with improved performance status, as defined by KPS-flag = 1. By using the nomogram, we intuitively derived a personalized risk assessment that corresponds to the KPS-flag.

An alternative approach to the nomogram we proposed could have been a nomogram that predicts KPS decline after surgery, as this could indeed have also significant clinical relevance, though additional data would be required to develop and validate such a model. In the present study, we decided to focus on the individuals who showed improvement in KPS because we wanted to concentrate our efforts on those who have a strong potential to continue the therapeutic process. By identifying this subgroup, we could better understand the factors that contributed to their progress and optimize resources for their continued support. In this context, further research is needed to examine the specific characteristics and factors of improvement, in order to determine whether specific demographic, clinical, or psychosocial factors played a role in their progress. Another issue is represented by the investigation of which aspects of the therapeutic process are most effective and whether certain treatment modalities or durations lead to better outcomes. Moreover, another important point that remains to be addressed is to investigate the sustainability of progress, particularly, whether improvements continue over time or whether some people regress, and which are the possible contributing factors. Finally, by understanding the barriers that may have prevented improvement in other individuals, the presented approach can be refined, allowing those making slower progress to also receive the support they need. Through this comprehensive evaluation, the effectiveness of therapeutic interventions can be improved, also allowing for the maximization of long-term success.

However, in this study, our modeling principle was a trade-off between a minimal number of features and the ability to make good predictions to avoid overfitting. In addition, to improve the performance of the prediction model, further studies with multiple ROIs are needed to ensure stability and reproducibility. Moreover, a comprehensive integrative analysis that includes molecular approaches could improve preoperative prognostic accuracy, enable personalized surgical planning, and improve patient counseling. In the future, a prospective multicenter study with a larger sample size is essential to refine predictive models for clinical application and overcome the inherent limitations of retrospective studies.

Furthermore, we recognize the importance of comparing AI-based models to traditional imaging assessments and clinical evaluations performed by experienced clinicians and radiologists. However, the aim of the present study is not to replace their expertise but rather to provide a supportive tool that enhances decision-making and assists in optimizing patient care. Finally, cost-effectiveness is a crucial factor for clinical implementation. AI-driven models primarily rely on standard imaging sequences (such as 3D T1 GRE), which are already part of routine clinical practice, minimizing additional imaging costs. The main expenses come from data processing, computational infrastructure, and the integration of AI into clinical workflows. If proven effective, such models could ultimately enhance decision-making, reduce unnecessary interventions, and improve patient outcomes, potentially justifying their costs in the long run. In this context, further studies are required to assess the exact economic impact and feasibility of widespread adoption in clinical settings.

## 5. Conclusions

In conclusion, the proposed ML approach using XGBoost was capable of defining radiomics features to predict the patients that potentially could improve their KPS after surgery. The interpretability obtained by our ML model allowed us to develop a multivariate logistic model defining a nomogram that can be used as a basis for further studies.

The future outlook for highlighting postoperative KPS in GG4 prognosis entails incorporating it into a comprehensive prognostic framework that includes molecular and imaging biomarkers.

## Figures and Tables

**Table 1:** The metrics obtained for the classification model. The metrics are computed both in cross-validation (CV) on the training set (mean with 95% confidence intervals) and in out-of-sample evaluations on the test set. ACC (accuracy) and MCC (Matthew’s correlation coefficient) are reported for both the CV and test evaluations. ACC: accuracy; CV (CI): cross-validation (confidence interval); RF: Random Forest.

Model	ACC CV (CI)	ACC Test	MCC CV (CI)	MCC Test
XGBoost	0.802 (0.797–0.806)	0.719	0.339 (0.330–0.3483)	0.302
RF	0.633 (0.56–0.701)	0.656	0.104 (−0.07–0.26)	0.120

**Table 2:** The logistic regression used to develop the clinical nomogram, providing a quantitative method for predicting outcomes based on selected clinical variables. The model estimates the odds ratio (OR) for each predictor, along with the corresponding 95% confidence interval (CI) and statistical significance (*p*-value) assessed using ANOVA. The OR quantifies the strength of association between each variable and the outcome, while the CI provides a range of plausible values, and the *p*-value indicates the statistical significance of each predictor’s contribution to the model. This approach enhances interpretability and facilitates individualized risk assessments. OR, odds ratio; CI, confidence interval; *p*-value, ANOVA *p*-value.

Characteristic	OR ^1^	95% CI ^1^	*p*-Value
Preoperative Volume	1.01	1.01 to 1.02	0.001
Age eldery			>0.99
<70	—	—	
>70	1.00	0.36 to 2.71	
Gender			0.62
Male	—	—	
Female	1.27	0.50 to 3.27	
Extent of Resection (EOR)	1.01	0.96 to 1.07	0.67
wavelet_hhh_glcm_correlation	0.32	0.14 to 0.69	0.003
localization			0.010
precentral	—	—	
postcentral	0.63	0.20 to 1.88	
temporoinsular	0.23	0.08 to 0.61	
log_sigma_4_0_mm_3d_firstorder_skewness	0.24	0.10 to 0.48	<0.001
wavelet_lll_firstorder_skewness	0.44	0.22 to 0.84	0.012
original_shape_elongation	1.65	1.08 to 2.60	0.021
wavelet_llh_firstorder_skewness	1.41	0.92 to 2.18	0.11
original_gldm_largedependencehighgraylevelemphasis	0.65	0.40 to 1.00	0.051
log_sigma_2_0_mm_3d_glcm_clustershade	1.62	0.93 to 2.88	0.088
wavelet_lhh_glcm_correlation	1.72	0.81 to 3.80	0.16
No. Obs.	157		
AIC	184		
BIC	230		

^1^ OR = odds ratio; CI = confidence interval.

## Data Availability

Data are available upon request to the corresponding author (Maurizio Polano). The data are not publicly available because they contain information that could compromise the privacy of research participants.

## Supplementary Materials

The following supporting information can be downloaded at: https://www.mdpi.com/article/10.3390/cancers17050758/s1. Figure S1: A flowchart of patient enrollment and data preprocessing for the eligibility criteria. (A) A total of 195 patients were initially enrolled, of whom 38 were excluded due to insufficient clinical or imaging data, leaving 157 eligible patients. These were then divided into a training cohort (125 patients) and a test cohort (32 patients) for model development and evaluation. (B) The data analysis protocol outlines the machine learning (ML) approach applied to extract and select predictive features. (C) The nomogram development protocol details the process of integrating selected features into a predictive model, aimed at facilitating individualized risk assessment and clinical decision-making. Figure S2: A comparison of the z-normal value of log_sigma_4_0_mm_3D_firstorder_skewness between two groups (improved KPS in blue and no improvement in red) within the Cystic/Necrotic subgroup. The boxplot represents the distribution of values, with individual data points superimposed as jittered points. A Wilcoxon rank sum test was performed to assess the statistical differences between the groups, and the corresponding *p*-value is displayed (*p* < 0.05). Figure S3: A Forest plot of the multivariate logistic model for the classification of patients with improved Karnofsky Performance Status (KPS). The figure illustrates the odds ratios (ORs) of each predictor variable included in the model. The blue dots represent the variables with ORs greater than 1, indicating an increased likelihood of KPS improvement (KPS-FLAG), while the corresponding confidence interval bars illustrate the statistical uncertainty around the estimate. In contrast, the red dots indicate variables with ORs less than 1, indicating a lower probability of KPS improvement. The horizontal line at OR = 1 serves as a reference, with values exceeding this threshold indicating no significant relationship. This visualization helps to identify the key factors influencing KPS outcomes and their relative contribution to patient classification. Figure S4: A calibration curve of the nomogram in the training and test cohorts. (A) A calibration curve of the training cohort nomogram. (B) A calibration curve of the validation cohort nomogram. The dashed line indicates the calibrated curve, the solid line indicates the original curve, and the diagonal line indicates the prediction model in an ideal state. The closer the curve shape is to the diagonal line, the better the prediction performance (x-axis: the predicted probability of a patient achieving a beneficial outcome; y-axis: the probability of an actual observed patient achieving a beneficial outcome). Figure S5. A decision curve analysis of the nomogram model. A decision curve analysis (DCA) to illustrate the net benefit of different predictive models for patient treatment decisions. The x-axis represents the threshold probability, while the y-axis shows the net benefit. The pure clinical model (blue), the pure radiographic model (yellow), and the combined nomogram model (red) are compared to evaluate their effectiveness. The net benefit takes into account the trade-off between the true and false positives and is used for optimal decision-making. The gray line represents the “treat all” strategy, where all patients are treated, while the black line represents the “treat none” strategy, where no patients are treated. The superior net benefit of the nomogram across a range of threshold probabilities suggests that it may be a more effective decision aid. Table S1: The basic clinical characteristics of the GG4 patients. Table S2: The association of significant variables with other clinical and biological prognosticators. Table S3: A comparison of the basic clinical characteristics between the train and test groups of the GG4 patients. Table S4: The selected features extracted from XGBoost. The selected features extracted from the XGBoost model and used for the development of univariate and multivariate logistic models. The table shows the characteristic variables identified by the FLAG-KPS model using XGBoost. These features are ordered by their influence on the model predictions and provide insight into the key factors that contribute to the performance of the model and the decision-making process [57,58,59,60,61,62,63].
