# Supplementary material for: Development of Clinical-Radiomics Nomogram for Predicting Post-Surgery Functional Improvement in High-Grade Glioma Patients
Source: Cancers (Basel). 2025 Feb 23;17(5):758. doi: 10.3390/cancers17050758 (PMC11899258; doi:10.3390/cancers17050758)
Supplement: Supplementary file 1 [file cancers-17-00758-s001.zip › cancers-3419186-supplementary.pdf]

**Description of additional information**

**Additional Methods**

**Additional Tables**

**Table S1.**

**Table S2**

**Table S3**

**Table S4**.

**Supplementary Figure Legends**

**Figure S1**

**Figure S2**

**Figure S3**

**Figure S4**

**Figure S5**

**CheckList for EvaluAtion of Radiomics research (CLEAR)**

## Additional Methods

### Machine learning approach

The data were split beforehand into 80% training and 20% test partitions. All models were developed in a 10× 5-fold cross validation (CV) schema on the training partition according to guidelines by the US FDA MicroArray/Sequencing Quality Control (MAQC/SEQC) initiatives [1–3]. For feature selection, the data were randomly split into 10 train and test partitions with an 80%/20% train/test proportion, preserving the original class stratification. Each of the 10 train partitions underwent 5 iterations of a stratified 5-fold cross-validation. A combination of two methods are used Pearson correlation matrix, a recursive feature elimination (RFE). The final dataset was created by integrating the list of the previous approach through the Borda algorithm[4]

The initial classification model was selected by evaluating the performance of an extreme gradient boosting (XGBoost) and Random Forrest (RF) in python 3.10 using xgboost [2] and sklearn library as previous described [6,7]. The optimal hyperparameters were selected with a grid search across a space of model-specific parameters using Hyperopt [4]. The search range for the hyper-parameters was $1 \times 10^{-5}$ to $1 \times 10^{-1}$ for the learning rate, 300 to 1000 for the number of trees, 1 to 4 for the minimum sum of instance weight needed in a child, 3 to 5 for maximum tree depth, 0.2 to 0.5 for the subsample ratio of the training instance, 0.2 to 0.5 for the subsample ratio of columns when constructing each tree, 0 to 0.1 for the minimum loss reduction, and 0 to 75 for L1 and L2 regularization terms on weights. Five-fold cross-validation was done on the training set to reduce overfitting. Performance was evaluated using accuracy (ACC), Area under the ROC curve (AUC) and the Matthews correlation coefficient (MCC) a performance metric that effectively summarizes the confusion matrix of a classification task into a single value, even when the classes are unbalanced. The MCC values are in the range [−1,1], where 1 indicates perfect classification, −1 indicates perfect misclassification, and 0

indicates random prediction or classification of each sample into the largest class. It has been shown that MCC is a more reliable metric than accuracy and F1 score[9]. The overall performance in cross-validation is evaluated across all CV iterations as average MCC and ACC with 95% Studentized bootstrap confidence intervals (CI), and on the test partition as MCC and ACC.

**Creation of the nomogram**

The C-index, AUC-ROC was calculated to assess the discrimination performance of the radiomics nomogram. In evaluating the calibration of the nomogram; we created a calibration curve that serves as a scatter plot and represents the probability of actual occurrence versus predictions. In addition, the results of the Hosmer-Lemeshow goodness-of-fit test were considered in the calibration assessment to provide a comprehensive assessment of the model's performance and reliability. The Hosmer-Lemeshow test was used to assess the fitness of the nomogram ($P > 0.05$ indicating good fit). The training and validation test were applied for all the models. Decision curve analysis (DCA) was used to investigate the clinical utility of the nomogram in both the training and validation sets by using rmda R package [10,11].

**Computational Details**

The classification pipeline was built on top of the Scikit Learn library 0.20.3 [3] using Python 3.10. All the experiments were run on a 32-core Intel Core i7 workstation with 128GB of RAM running Ubuntu 22. Statistical analyses were computed using R (version 4.3.1).

**References**

1. Shi L, Campbell G, Jones WD, Campagne F, Wen Z, Walker SJ, et al. The MicroArray Quality Control (MAQC)-II study of common practices for the development and validation of microarray-based predictive models. Nat Biotechnol. 2010;28:827–38.

2. Chiesa S, Russo R, Beghella Bartoli F, Palumbo I, Sabatino G, Cannatà MC, et al. MRI-derived radiomics to guide post-operative management of glioblastoma: Implication for personalized radiation treatment volume delineation. Front Med. 2023;10:1059712.

3. Ren J, Zhai X, Yin H, Zhou F, Hu Y, Wang K, et al. Multimodality MRI Radiomics Based on Machine Learning for Identifying True Tumor Recurrence and Treatment-Related Effects in Patients with Postoperative Glioma. Neurol Ther. 2023;12:1729–43.

4. Burka D, Puppe C, Szepesváry L, Tasnádi A. Voting: A machine learning approach. Eur J Oper Res. 2022;299:1003–17.

5. Chen T, Guestrin C. XGBoost: A Scalable Tree Boosting System. Proc 22nd ACM SIGKDD Int Conf Knowl Discov Data Min [Internet]. New York, NY, USA: Association for Computing Machinery; 2016 [cited 2024 Feb 26]. p. 785–94. Available from: https://dl.acm.org/doi/10.1145/2939672.2939785

6. Polano M, Chierici M, Dal Bo M, Gentilini D, Di Cintio F, Baboci L, et al. A Pan-Cancer Approach to Predict Responsiveness to Immune Checkpoint Inhibitors by Machine Learning. Cancers. 2019;11.

7. Pedregosa F, Varoquaux G, Gramfort A, Michel V, Thirion B, Grisel O, et al. Scikit-learn: Machine Learning in Python. J Mach Learn Res. 2011;12:2825–30.

8. Bergstra J, Yamins D, Cox D. Making a Science of Model Search: Hyperparameter Optimization in Hundreds of Dimensions for Vision Architectures. Proc 30th Int Conf Mach Learn [Internet]. PMLR; 2013 [cited 2024 Feb 8]. p. 115–23. Available from: https://proceedings.mlr.press/v28/bergstra13.html

9. Chicco D, Jurman G. The advantages of the Matthews correlation coefficient (MCC) over F1 score and accuracy in binary classification evaluation. BMC Genomics. 2020;21:6.

10. Vickers AJ, Cronin AM, Elkin EB, Gonen M. Extensions to decision curve analysis, a novel method for evaluating diagnostic tests, prediction models and molecular markers. BMC Med Inform Decis Mak. 2008;8:53.

11. Vickers AJ, Elkin EB. Decision curve analysis: a novel method for evaluating prediction models. Med Decis Mak Int J Soc Med Decis Mak. 2006;26:565–74.

**Table S1.** The basic clinical characteristics of the GG4 patients

| Characteristic | N = 157[1] |
|---|---|
| Age at surgery | 61 (53 – 70) |
| Gender | |
| Male | 106 (68) |
| Female | 51 (32) |
| localization | |
| precentral | 51 (32) |
| postcentral | 38 (24) |
| temporoinsular | 68 (43) |
| side | |
| left | 77 (49) |
| right | 80 (51) |
| IDH status | |
| wt | 140 (89) |
| mut | 17 (11) |
| MGMT methylation status | |
| unmethylated | 62 (39) |
| methylated | 95 (61) |
| Preoperative volume | 76 (43 – 126) |
| KPS-PRE | |
| 60 | 3 (1.9) |
| 70 | 23 (15) |
| 80 | 48 (31) |
| 90 | 30 (19) |
| 100 | 53 (34) |
| KPS-POST | |

| **Characteristic** | **N = 157**[1] |
|---|---|
| 50 | 1 (0.6) |
| 60 | 6 (3.8) |
| 70 | 15 (9.6) |
| 80 | 34 (22) |
| 90 | 26 (17) |
| 100 | 75 (48) |
| MRI index | 0.46 (0.26 – 0.65) |
| FLAG-KPS | |
| 0 | 102 (65) |
| 1 | 55 (35) |
| Hypertension | |
| 0 | 87 (55) |
| 1 | 70 (45) |
| Ependyma involvement | |
| No | 127 (81) |
| Yes | 30 (19) |
| Necrotic/Cystic | |
| No | 37 (24) |
| Yes | 120 (76) |
| Midline Shift | |
| No | 81 (52) |
| Yes | 76 (48) |
| Radiotherapy | |
| No | 3 (1.9) |
| Yes | 154 (98) |
| Chemotherapy | |

| Characteristic | N = 157[1] |
|---|---|
| No | 10 (6.4) |
| Yes | 147 (94) |
| hospital length of stay | 10.0 (8.0 – 15.0) |
| Extent of Resection (EOR) | 98 (95 – 100) |
| Second Surgery | |
| No | 134 (85) |
| Yes | 23 (15) |
| Complete Stupp | |
| 0 | 11 (7.0) |
| 1 | 146 (93) |
| Age eldery | |
| <=70 | 120 (76) |
| >70 | 37 (24) |
| Extent of Resection categorical | |
| < 100% EOR | 88 (56) |
| =100% EOR | 69 (44) |
| KPS-POST categorical | |
| KPS POST< 90 | 82 (52) |
| KPS POST > 90 | 75 (48) |

[1]Median (IQR); n (%)

**Table S2.** The Association of significant variables with other clinical and biological prognosticators

| Variables | MGMT STATUS | | p values |
|---|---|---|---|
| | Unmethylated N=62 | Methylated N=95 | |
| Extent of Resection (EOR) | 95 (90, 100) | 98 (95, 100) | **0.042** |
| **Variables** | **IDH STATUS** | | **p values** |
| | wildtype N=140 | mut N=17 | |
| Preoperative Volume | 78 (45, 127) | 43 (32, 64) | **0.043** |
| Age at surgery | 63 (54, 70) | 53 (46, 56) | **0.001** |
| Complete Stupp | | | |
| No | 10 (7.1%) | 17 (100%) | **<0.001** |
| Yes | 130 (93%) | 0 (0%) | |
| **Variables** | **GENDER** | | **p values** |
| | Male, N=106 | Female N=51 | |
| localization | | | 0.001 |
| precentral | 26 (25%) | 25 (49%) | |
| postcentral | 24 (23%) | 14 (27%) | |
| temporoinsular | 56 (53%) | 12 (24%) | |

| | | | | |
|---|---|---|---|---|
| Preoperative Volume | 78 (45, 132) | | 70 (30, 110) | 0.036 |
| **Variables** | **LOCALIZATION** | | | **p values** |
| | **precentral**, N = 51 | **postcentral**, N = 38 | **temporoinsular**, N = 68 | |
| Gender | | | | 0.001 |
| Male | 26 (51%) | 24 (63%) | 56 (82%) | |
| Female | 25 (49%) | 14 (37%) | 12 (18%) | |
| Preoperative Volume | 78 (48, 132) | 46 (32, 107) | 84 (52, 132) | 0.011 |
| Ependyma involvement | | | | <0.001 |
| No | 31 (61%) | 31 (82%) | 65 (96%) | |
| Yes | 20 (39%) | 7 (18%) | 3 (4.4%) | |
| Midline Shift | | | | 0.004 |
| No | 28 (55%) | 27 (71%) | 26 (38%) | |
| Yes | 23 (45%) | 11 (29%) | 42 (62%) | |

**Table S3.** Comparison of the basic clinical characteristics between Train and Test Groups of GG4 Patients.

| **Characteristic** | **Train data** N = 125[1] | **Test data** N = 32[1] | **p-value**[2] |
|---|---|---|---|
| Age at surgery | 61 (53, 70) | 62 (54, 69) | >0.9 |
| IDH Status | | | 0.8 |
| wt | 112 (90%) | 28 (88%) | |
| mut | 13 (10%) | 4 (13%) | |
| *KPS-flag* | | | >0.9 |
| 0 | 81 (65%) | 21 (66%) | |
| 1 | 44 (35%) | 11 (34%) | |
| MGMT methylation status | | | 0.9 |
| Unmethylated | 49 (39%) | 13 (41%) | |
| methylated | 76 (61%) | 19 (59%) | |
| Volume pre T2 | 77 (42, 123) | 73 (45, 130) | 0.7 |
| Side | | | 0.8 |
| left | 62 (50%) | 15 (47%) | |
| right | 63 (50%) | 17 (53%) | |
| localization | | | 0.3 |
| precentral | 44 (35%) | 7 (22%) | |
| postcentral | 28 (22%) | 10 (31%) | |
| temporoinsular | 53 (42%) | 15 (47%) | |
| Extent of Resection categorical | | | 0.4 |
| < 100% EOR | 72 (58%) | 16 (50%) | |
| =100% EOR | 53 (42%) | 16 (50%) | |
| Delta_T1vsT2 | 0.46 (0.27, 0.66) | 0.52 (0.18, 0.63) | >0.9 |

| **Characteristic** | **Train data** N = 125[1] | **Test data** N = 32[1] | **p-value**[2] |
|---|---|---|---|
| Extent of Resection (EOR) | 98 (95, 100) | 99 (95, 100) | 0.4 |
| Gender | | | 0.3 |
| Male | 82 (66%) | 24 (75%) | |
| Female | 43 (34%) | 8 (25%) | |

[1]Median (IQR); n (%),[2]Wilcoxon rank sum test; Fisher's exact test; Pearson's Chi-squared test

**Table S4**. Selected Features Extracted from XGBoost. Selected features extracted from the XGBoost model and used for the development of univariate and multivariate logistic models. The table shows the characteristic variables identified by the FLAG-KPS model using XGBoost. These features are ordered by their influence on the model predictions and provide insight into the key factors that contribute to the performance of the model and the decision-making process.

| |
|---|
| log-sigma-4-0-mm-3d_firstorder_skewness |
| log-sigma-4-0-mm-3d_firstorder_maximum |
| log-sigma-3-0-mm-3d_firstorder_skewness |
| wavelet-hhh_firstorder_skewness |
| wavelet-hhl_glcm_clustershade |
| wavelet-hhh_glcm_correlation |
| wavelet-lll_glcm_imc2 |
| wavelet-lhh_glcm_imc2 |
| wavelet-lhl_glcm_imc2 |
| wavelet-hlh_glcm_clustershade |
| wavelet-lhl_firstorder_maximum |
| wavelet-hhl_glcm_imc1 |
| log-sigma-5-0-mm-3d_firstorder_skewness |
| wavelet-hhh_glcm_imc1 |
| wavelet-hhl_glszm_smallareaemphasis |
| wavelet-hhl_glcm_correlation |
| original_shape_elongation |
| original_gldm_largedependencehighgraylevelemphasis |
| wavelet-lhl_glszm_smallareaemphasis |
| log-sigma-3-0-mm-3d_firstorder_maximum |
| wavelet-lhl_glszm_zoneentropy |
| original_glszm_lowgraylevelzoneemphasis |

| |
|---|
| log-sigma-3-0-mm-3d_glcm_clustershade |
| wavelet-hhh_glszm_smallareaemphasis |
| wavelet-hlh_firstorder_median |
| wavelet-hlh_glcm_correlation |
| wavelet-hlh_glcm_imc2 |
| wavelet-lhh_firstorder_median |
| wavelet-lll_firstorder_variance |
| wavelet-hlh_glcm_idmn |
| wavelet-lll_firstorder_skewness |
| wavelet-hhl_firstorder_skewness |
| wavelet-lll_glszm_lowgraylevelzoneemphasis |
| original_firstorder_maximum |
| wavelet-hhl_gldm_smalldependencelowgraylevelemphasis |
| wavelet-llh_glcm_clustershade |
| original_shape_sphericity |
| wavelet-lhl_gldm_dependenceentropy |
| wavelet-llh_firstorder_skewness |
| wavelet-hhh_firstorder_mean |
| wavelet-lhh_glcm_correlation |
| wavelet-lhl_glcm_clustershade |
| wavelet-hll_glcm_imc2 |
| log-sigma-2-0-mm-3d_glcm_clustershade |

**Supplementary Figure Legends**

**Figure S1.** Flowchart of patient enrollment and data preprocessing for eligibility criteria. (A) A total of 195 patients were initially enrolled, of whom 38 were excluded due to insufficient clinical or imaging data, leaving 157 eligible patients. These were then divided into a training cohort (125 patients) and a test cohort (32 patients) for model development and evaluation. (B) The data analysis protocol outlines the machine learning (ML) approach applied to extract and select predictive features. (C) The nomogram development protocol details the process of integrating selected features into a predictive model, aimed at facilitating individualized risk assessment and clinical decision-making.

**Figure S2.** Comparison of z-normal value of log_sigma_4_0_mm_3D_firstorder_skewness between two groups (improved KPS in blue and no improvement in red) within the cystic/necrotic subgroup. The boxplot represents the distribution of values, with individual data points superimposed as jittered points. A Wilcoxon rank sum test was performed to assess the statistical differences between the groups and the corresponding p-value is displayed ($p<0.05$).

**Figure S3.** Forest plot of the multivariate logistic model for the classification of patients with improved Karnofsky Performance Status (KPS). The figure illustrates the odds ratios (ORs) of each predictor variable included in the model. Blue dots represent variables with ORs greater than 1, indicating an increased likelihood of KPS improvement (KPS-FLAG), while the corresponding confidence interval bars illustrate the statistical uncertainty around the estimate. In contrast, red dots indicate variables with ORs less than 1, indicating a lower probability of KPS improvement. The horizontal line at OR = 1 serves as a reference, with values exceeding

this threshold indicating no significant relationship. This visualization helps to identify the key factors influencing KPS outcomes and their relative contribution to patient classification.

**Figure S4.** Calibration curve of the nomogram in the training and test cohorts. A) Calibration curve of training cohort nomogram, B). Calibration curve of validation cohort nomogram. The dashed line indicates the calibrated curve, the solid line indicates the original curve, the diagonal line indicates the prediction model in an ideal state. The closer the curve shape is to the diagonal line indicates better prediction performance. (x-axis: the predicted probability of a patient achieving a beneficial outcome; y-axis: the probability of an actual observed patient achieving a beneficial outcome)

**Figure S5. Decision curve analysis of nomogram model.** Decision curve analysis (DCA) to illustrate the net benefit of different predictive models for patient treatment decisions. The x-axis represents the threshold probability, while the y-axis shows the net benefit. The pure clinical model (blue), the pure radiographic model (yellow) and the combined nomogram model (red) are compared to evaluate their effectiveness. The net benefit takes into account the trade-off between true and false positives and is used for optimal decision making. The gray line represents the 'treat all' strategy, where all patients are treated, while the black line represents the 'treat none' strategy, where no patients are treated. The superior net benefit of the nomogram across a range of threshold probabilities suggests that it may be a more effective decision aid.

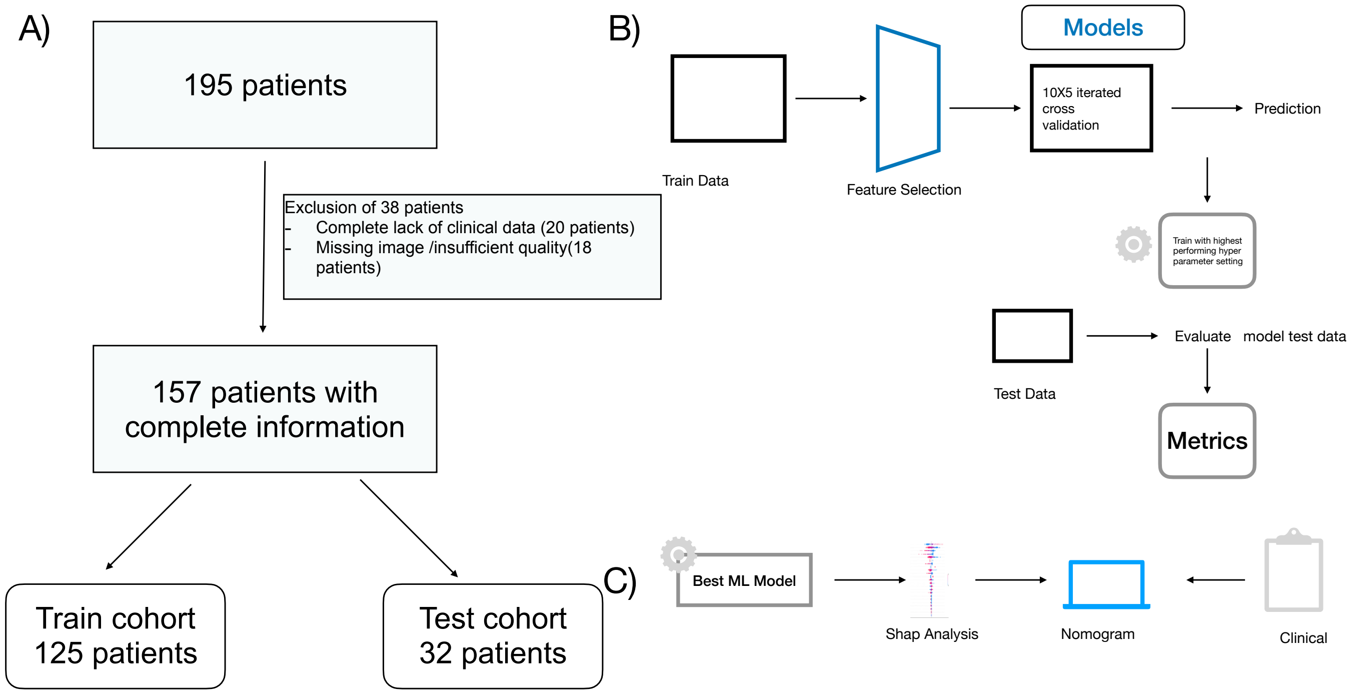
A)
195 patients
Exclusion of 38 patients
- Complete lack of clinical data (20 patients)
- Missing image /insufficient quality(18 patients)
157 patients with complete information
Train cohort 125 patients
Test cohort 32 patients
B)
Models
Train Data
Feature Selection
10X5 iterated cross validation
Prediction
Train with highest performing hyper parameter setting
Test Data
Evaluate model test data
Metrics
C)
Best ML Model
Shap Analysis
Nomogram
Clinical

Figure S1

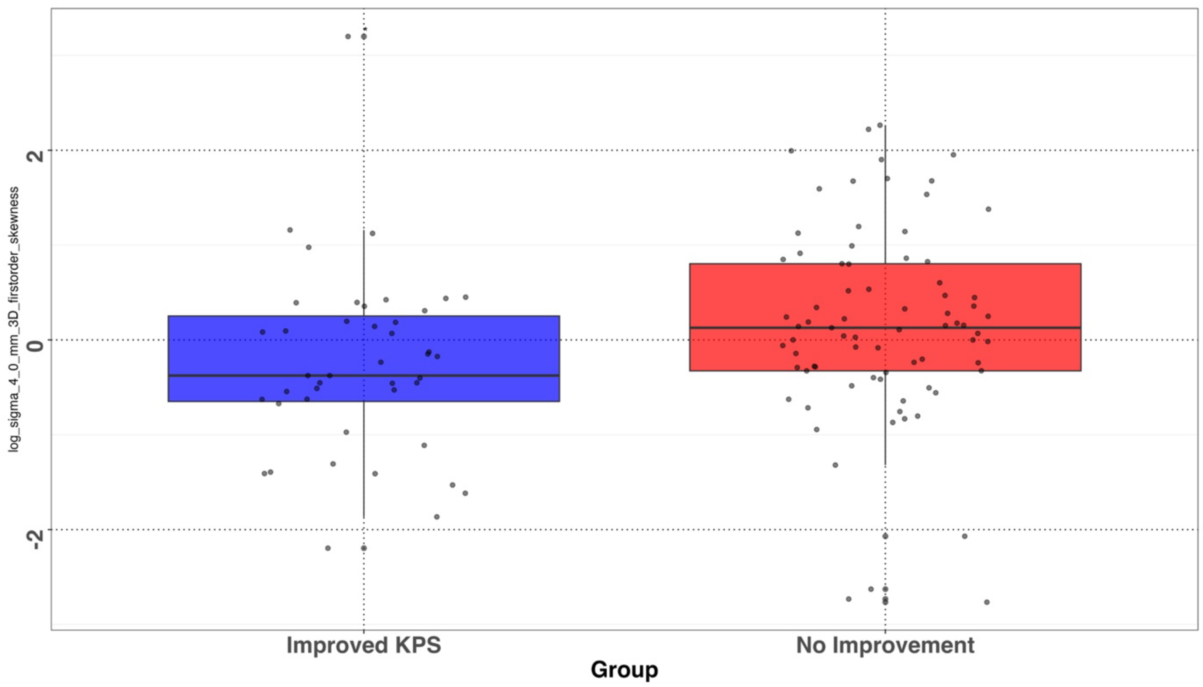
log_sigma_4_0_mm_3D_firstorder_skewness
2
0
-2
Improved KPS
No Improvement
Group

**Figure S2**

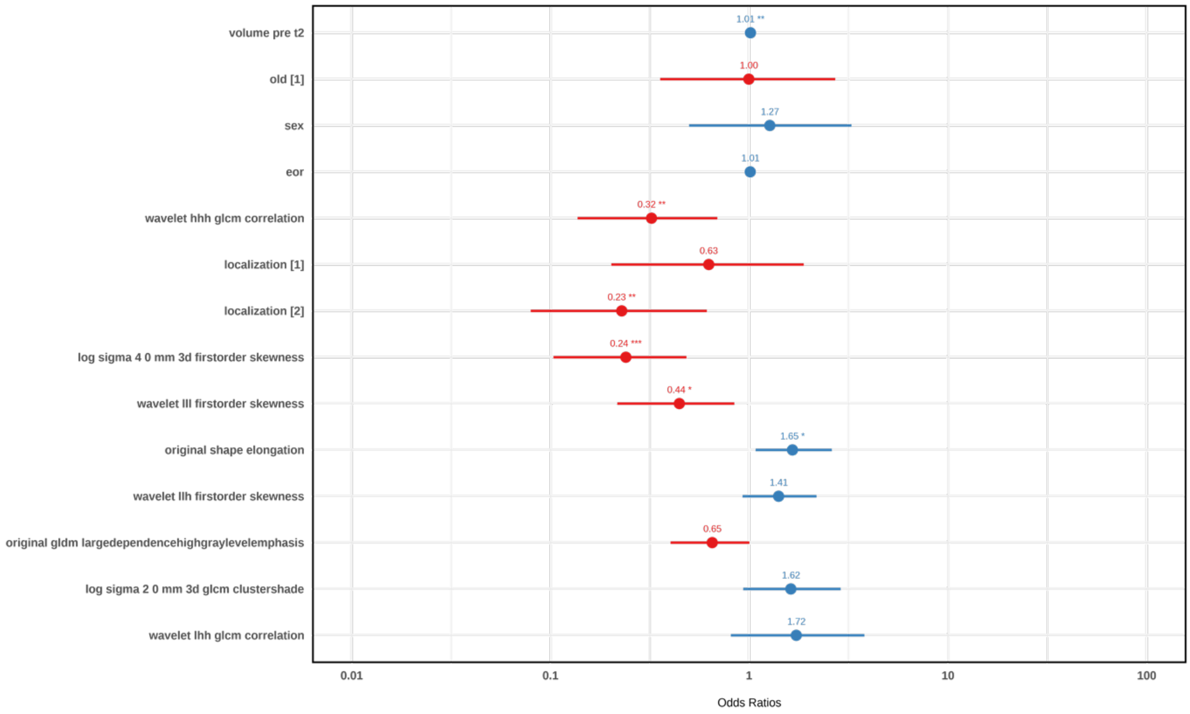
volume pre t2
old [1]
sex
eor
wavelet hhh glcm correlation
localization [1]
localization [2]
log sigma 4 0 mm 3d firstorder skewness
wavelet lll firstorder skewness
original shape elongation
wavelet llh firstorder skewness
original gldm largedependencehighgraylevelemphasis
log sigma 2 0 mm 3d glcm clustershade
wavelet lhh glcm correlation
1.01 **
1.00
1.27
1.01
0.32 **
0.63
0.23 **
0.24 ***
0.44 *
1.65 *
1.41
0.65
1.62
1.72
0.01
0.1
1
10
100
Odds Ratios

**Figure S3**

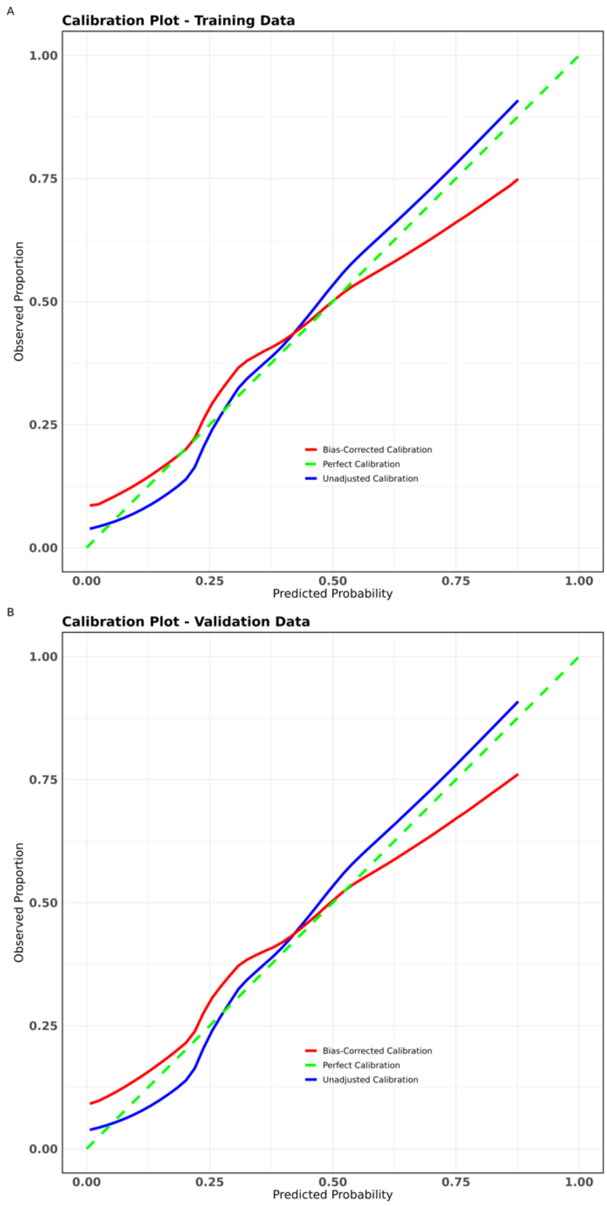
A
Calibration Plot - Training Data
1.00
0.75
0.50
0.25
0.00
Observed Proportion
Bias-Corrected Calibration
Perfect Calibration
Unadjusted Calibration
0.00
0.25
0.50
0.75
1.00
Predicted Probability
B
Calibration Plot - Validation Data
1.00
0.75
0.50
0.25
0.00
Observed Proportion
Bias-Corrected Calibration
Perfect Calibration
Unadjusted Calibration
0.00
0.25
0.50
0.75
1.00
Predicted Probability

**Figure S4**

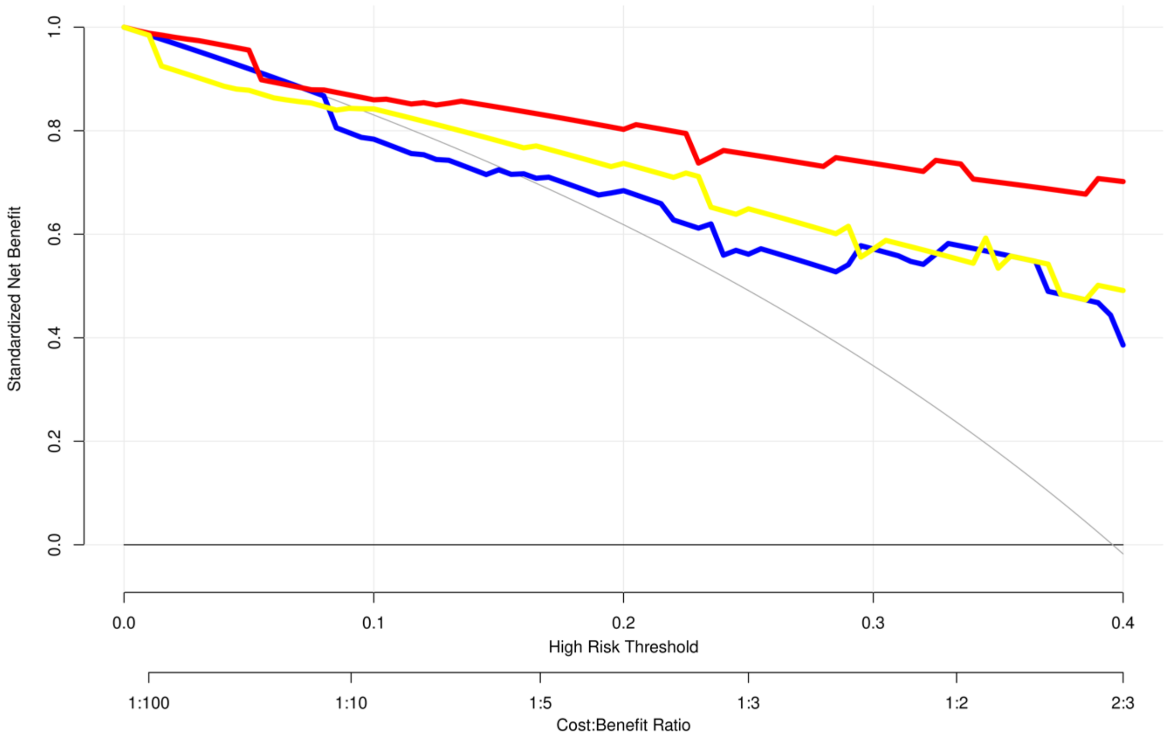
1.0
0.8
0.6
0.4
0.2
0.0
Standardized Net Benefit
0.0
0.1
0.2
0.3
0.4
High Risk Threshold
1:100
1:10
1:5
1:3
1:2
2:3
Cost:Benefit Ratio

**Figure S5**

# CLEAR Checklist v1.0

**Note**: Use the checklist in conjunction with the main text for clarification of all items.
Yes, details provided; No, details not provided; n/e, not essential; n/a, not applicable; Page, page number

| Section | No. | Item | Yes | No | n/a | Page |
|---|---|---|---|---|---|---|
| **Title** | | | | | | |
| | 1 | Relevant title, specifying the radiomic methodology | ☑ | ☐ | ☐ | 1 |
| **Abstract** | | | | | | |
| | 2 | Structured summary with relevant information | ☑ | ☐ | ☐ | 2 |
| **Keywords** | | | | | | |
| | 3 | Relevant keywords for radiomics | ☑ | ☐ | ☐ | 2 |
| **Introduction** | | | | | | |
| | 4 | Scientific or clinical background | ☑ | ☐ | ☐ | 3-4 |
| | 5 | Rationale for using a radiomic approach | ☐ | ☐ | ☐ | 3-4 |
| | 6 | Study objective(s) | ☐ | ☐ | ☐ | 4 |
| **Method** | | | | | | |
| *Study design* | 7 | Adherence to guidelines or checklists (e.g., CLEAR checklist) | ☑ | ☐ | ☐ | 5 |
| | 8 | Ethical details (e.g., approval, consent, data protection) | ☐ | ☐ | ☐ | 5 |
| | 9 | Sample size calculation | ☐ | ☑ | ☐ | |
| | 10 | Study nature (e.g., retrospective, prospective) | ☑ | ☐ | ☐ | 5,9 |
| | 11 | Eligibility criteria | ☑ | ☐ | ☐ | |
| | 12 | Flowchart for technical pipeline | ☐ | ☑ | ☐ | |
| *Data* | 13 | Data source (e.g., private, public) | ☑ | ☐ | ☐ | 5-6 |
| | 14 | Data overlap | ☐ | ☑ | ☐ | |
| | 15 | Data split methodology | ☑ | ☐ | ☐ | 5-7,A |
| | 16 | Imaging protocol (i.e., image acquisition and processing) | ☑ | ☐ | ☐ | 6 |
| | 17 | Definition of non-radiomic predictor variables | ☑ | ☐ | ☐ | 5-7 |
| | 18 | Definition of the reference standard (i.e., outcome variable) | ☑ | ☐ | ☐ | 5 |
| *Segmentation* | 19 | Segmentation strategy | ☑ | ☐ | ☐ | 6 |
| | 20 | Details of operators performing segmentation | ☑ | ☐ | ☐ | 6 |
| *Pre-processing* | 21 | Image pre-processing details | ☑ | ☐ | ☐ | 6 |
| | 22 | Resampling method and its parameters | ☑ | ☐ | ☐ | 6 |
| | 23 | Discretization method and its parameters | ☑ | ☐ | ☐ | 6 |

| Section | No. | Item | Yes | No | n/a | Page |
|---|---|---|---|---|---|---|
| | 24 | Image types (e.g., original, filtered, transformed) | ☑ | ☐ | ☐ | 6 |
| *Feature extraction* | 25 | Feature extraction method | ☑ | ☐ | ☐ | 6 |
| | 26 | Feature classes | ☑ | ☐ | ☐ | 6-7,T |
| | 27 | Number of features | ☑ | ☐ | ☐ | 6,9,1 |
| | 28 | Default configuration statement for remaining parameters | ☑ | ☐ | ☐ | 6 |
| *Data preparation* | 29 | Handling of missing data | ☑ | ☐ | ☐ | 8 |
| | 30 | Details of class imbalance | ☑ | ☐ | ☐ | 8-10, |
| | 31 | Details of segmentation reliability analysis | ☐ | ☐ | ☐ | 6-12 |
| | 32 | Feature scaling details (e.g., normalization, standardization) | ☑ | ☐ | ☐ | 6,Ad |
| | 33 | Dimension reduction details | ☑ | ☐ | ☐ | Addit |
| *Modeling* | 34 | Algorithm details | ☑ | ☐ | ☐ | Addit |
| | 35 | Training and tuning details | ☑ | ☐ | ☐ | Addit |
| | 36 | Handling of confounders | ☑ | ☐ | ☐ | Addit |
| | 37 | Model selection strategy | ☑ | ☐ | ☐ | Addit |
| *Evaluation* | 38 | Testing technique (e.g., internal, external) | ☑ | ☐ | ☐ | Addit |
| | 39 | Performance metrics and rationale for choosing | ☑ | ☐ | ☐ | 6:9,A |
| | 40 | Uncertainty evaluation and measures (e.g., confidence intervals) | ☑ | ☐ | ☐ | 6:9,A |
| | 41 | Statistical performance comparison (e.g., DeLong's test) | ☑ | ☐ | ☐ | 6:9,A |
| | 42 | Comparison with non-radiomic and combined methods | ☐ | ☐ | ☐ | 6:9,A |
| | 43 | Interpretability and explainability methods | ☐ | ☑ | ☐ | |
| **Results** | | | | | | |
| | 44 | Baseline demographic and clinical characteristics | ☑ | ☐ | ☐ | 9 |
| | 45 | Flowchart for eligibility criteria | ☐ | ☑ | ☐ | |
| | 46 | Feature statistics (e.g., reproducibility, feature selection) | ☐ | ☐ | ☐ | |
| | 47 | Model performance evaluation | ☑ | ☐ | ☐ | 9:12 |
| | 48 | Comparison with non-radiomic and combined approaches | ☐ | ☐ | ☑ | |
| **Discussion** | | | | | | |
| | 49 | Overview of important findings | ☑ | ☐ | ☐ | 13,14 |
| | 50 | Previous works with differences from the current study | ☑ | ☐ | ☐ | 13,14 |
| | 51 | Practical implications | ☐ | ☐ | ☐ | 13,14 |
| | 52 | Strengths and limitations (e.g., bias and generalizability issues) | ☑ | ☐ | ☐ | 13,14 |

| Section | No. | Item | Yes | No | n/a | Page |
|---|---|---|---|---|---|---|
| **Open Science** | | | | | | |
| *Data availability* | 53 | Sharing images along with segmentation data [n/e] | ☐ | ☑ | ☐ | |
| | 54 | Sharing radiomic feature data | ☐ | ☐ | ☑ | |
| *Code availability* | 55 | Sharing pre-processing scripts or settings | ☐ | ☑ | ☐ | |
| | 56 | Sharing source code for modeling | ☐ | ☑ | ☐ | |
| *Model availability* | 57 | Sharing final model files | ☐ | ☑ | ☐ | |
| | 58 | Sharing a ready-to-use system [n/e] | ☑ | ☐ | ☐ | shyna |

**Kocak B, Baessler B, Bakas S, Cuocolo R, Fedorov A, Maier-Hein L, Mercaldo N, Müller H, Orlhac F, Pinto Dos Santos D, Stanzione A, Ugga L, Zwanenburg A. CheckList for EvaluAtion of Radiomics research (CLEAR): a step-by-step reporting guideline for authors and reviewers endorsed by ESR and EuSoMII. Insights Imaging. 2023 May 4;14(1):75. doi: 10.1186/s13244-023-01415-8**
